# Supplementary material for: Transformable topological mechanical metamaterials
Source: Nat Commun. 2017 Jan 23;8:14201. doi: 10.1038/ncomms14201 (PMC5264209; doi:10.1038/ncomms14201)
Supplement: Supplementary Information — Supplementary Figures, Supplementary Notes and Supplementary References [file ncomms14201-s1.pdf]

SUPPLEMENTARY NOTE 1: INFORMATION ON THE VIDEO DEMONSTRATION

The prototype is constructed of commercially available plastic "K'NEX" parts. A rigid triangle consists of three rods extending from a central white connector. There are two species of triangles of different shapes (red and blue, as shown in Fig. 1): those ending in blue hinge-parts and those ending in black hinge-parts. Note that although there are no direct connections between two hinge-parts in the same triangle the length between them is fixed by the rods joining them to the central part (which cannot rotate relative to one another) so the triangles are rigid. Each pair of blue hinge-part and black hinge-part form one flexible hinge. Connected triangles are thus able to rotate freely relative to one another. This is a realization of the deformed kagome lattice described in the main text.

The frame consists of four metal rods connected to triangles on the edge of the structure and manipulated by hand. The triangles are free to slide along the lengths of the rods so that the spacing between edge triangles changes even as they remain collinear. The rods are rotated relative to one another, resulting in a uniform soft twisting as described in the main text that alters the lattice structure of the prototype.

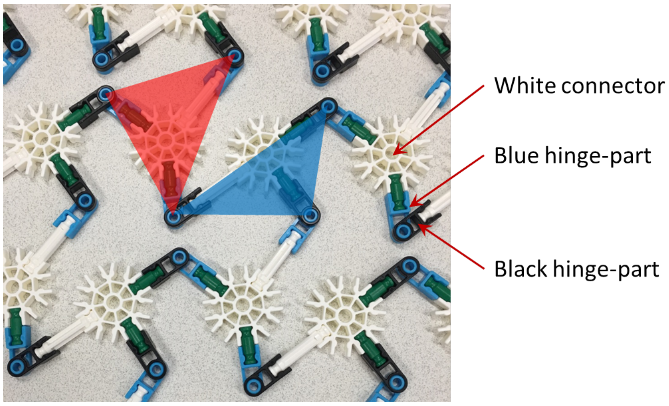

Supplementary Figure 1. **The plastic prototype used in the video demonstrations.**

SUPPLEMENTARY NOTE 2: GENERALIZED MAXWELL'S COUNTING RULE AND UNIFORM SOFT TWISTINGS

The number of zero modes (modes of deformation which cost no energy) N_z of a structure is determined by the numbers of degrees of freedom $N_{\text{d.o.f.}}$, constraints N_c and states of self stress (i.e., possible ways to distribute internal stress without net forces on any parts) N_{ss} through the generalized Maxwell's counting rule [2, 3]

$$N_z = N_{\text{d.o.f.}} - N_c + N_{ss}. \tag{1}$$

One simple setup to demonstrate this relation is a frame consisting of N_c struts connected at N free hinges (e.g., the structure in Fig. 1b in the Article). For a system with spatial dimension d, each hinge needs a d-component coordinate to describe its location, so it has d degrees of freedom and $N_{\text{d.o.f.}} = Nd$. Each strut fixes the distance between two hinges and thus enforces one constraint. It is worthwhile to note that the constraints enforced by struts may not be independent, i.e., some of the struts may be redundant and thus do not introduce new constraints. As shown in Ref. [3], each redundant constraint contributes one state of self-stress (i.e., stress may be introduced if the length of the strut change), which is the last term in Eq. (1). The term isostatic refers to the special marginal state where $N_z = d(d+1)/2$ (only trivial zero modes corresponding to rigid translations and rotations of the whole system exist) and $N_{ss} = 0$ where the structure is both stable and stress-free. A critical mean coordination number $\langle z \rangle = 2d$ for isostaticity [4–6] follows from $N_{\text{d.o.f.}} = N_c$, which is a weaker condition of mechanical stability that assumes all struts are independent. Following the nomenclature of Ref. [7] we call periodic lattices with $\langle z \rangle = 2d$ "Maxwell lattices".

When the generalized Maxwell's counting rule is applied to periodic lattices, as shown in Refs. [7, 8], an interesting consequence follows that all lattices with $\langle z \rangle = 2d$ (Maxwell lattices) must have $d(d-1)/2$ homogeneous deformations that are of zero energy. For 2D lattices, the case this Article is mainly concerned with, Maxwell lattices have at least one such soft deformation (which we name the uniform soft twisting). These floppy modes have also been called "Guest modes" [7, 8].

Certain lattices with $\langle z \rangle > 2d$, such as the deformed checkerboard lattice in Fig. 2 in the Article, also possess uniform soft twistings, with these necessarily accompanied by states of self stress.

In addition, this type of counting rules and the resulting floppy deformations apply equally to simple frames with struts-hinges and more complicated structures, provided that the degrees of freedom and constraints are countable. For example, a sub-class of these floppy deformations, the "rigid-unit-modes" (RUMs), has been studied in the context of crystals with the structure of periodic corner-touching polyhedra and argued to be responsible for negative thermal expansion in some crystals [9, 10], as well as utilized to realize negative Poisson's ratio metamaterials [11, 12]. In this Article we discuss more general situations which do not necessarily involve rigid polyhedra.

SUPPLEMENTARY NOTE 3: NUMERICAL CALCULATION OF EDGE STIFFNESS

Systems of 60 × 60 unit cells were generated. Three of the four sides were held fixed, while one triangle from the free side was pressed into the structure in the linear regime (qualitatively similar behavior was observed under nonlinear deformations). The Conjugate Gradient method was used to obtain the minimum-energy configuration and the ratio of force to displacement was extracted as the edge stiffness. Units were chosen such that the spring constant of the struts and the length of the strut that is horizontal in Fig. 1a (in the Article) were both unity.

The residual edge stiffness of the soft edge is due to finite size effects as the sides of the lattice are clamped. Because the zero modes are exponentially localized to the soft edge, the stiffness of this edge falls exponentially with system size. In real systems this soft edge stiffness will be controlled by friction or bending stiffness at the hinges. In addition, the sharp rise in the edge stiffness of the soft edge at θ_3 is due to the fine-tuned geometrical effect of the line of struts being pulled taut in the transverse direction.

SUPPLEMENTARY NOTE 4: ELASTICITY THEORY OF UNIFORM SOFT TWISTINGS AND FLOPPY MODES

A. Elastic deformations and the strain tensor

In order to provide a self-contained discussion, here we first briefly review some basic concepts on elasticity.

In an elastic system, if we focus on macroscopic phenomena at length scales much longer than the scale of the microscopic structure, we can ignore microscopic details and treat the system as a continuous medium. In such a picture, each point in the elastic medium can be labeled by its coordinate $\mathbf{r}$ (here we use bold symbols to represent vectors and tensors). Under deformation, the point $\mathbf{r}$ is now displaced to a new location with coordinate $\mathbf{R}$. Such a deformation is described by a mapping $\mathbf{r} \to \mathbf{R}(\mathbf{r})$. In this language, the space that $\mathbf{r}$ lives in is called the reference space, i.e., the space before the deformation. and the space that $\mathbf{R}$ lives in is dubbed the target space, i.e. the space after deformation.

For a slowly varying displacement field, one can keep only the first order derivative $\partial_i R_j = \frac{\partial R_j}{\partial r_i}$ (where i, j are Cartesian indices denoting x, y in 2D) in the elastic energy and ignore higher order derivatives. This derivative, $\partial_i R_j$, appears to be a rank-2 tensor. However, it is important to realize that the two indices of this matrix live in two different spaces. The index i is from $\mathbf{r}$, which lives in the reference space, but the other index j is from $\mathbf{R}$, which lives in the target space. Symmetry transformations are independent in these two spaces (e.g., a rotation before deformation and the same rotation after deformation result in different strains of the elastic medium). To express the strain field as a true tensor one can contract either the reference space or the target space indices. A convenient choice is the metric tensor

$$g_{ij} = \partial_i R_k \partial_j R_k, \tag{2}$$

which is a tensor that lives in the reference space (i, j here are both indices in the reference space, and indices in the target space are contracted). Here we follow the Einstein summation convention, i.e. the repeated index k is summed over.

It is easy to verify if there is no deformation, $\mathbf{R}(\mathbf{r}) = \mathbf{r}$ up to rigid translations and rotations, the metric tensor is the identity matrix. To describe the strain, the left Cauchy Green strain tensor is defined by subtracting the identity matrix from the metric tensor,

$$\epsilon_{ij} = \frac{1}{2}(g_{ij} - \delta_{ij}), \tag{3}$$

where δ represents an identity matrix ($\delta_{ij} = 1$ for $i = j$ and $\delta_{ij} = 0$ otherwise).

B. Elastic energy and zero energy deformations

In this section, we prove that if there exists one uniform deformation that does not cost any elastic energy, the system must also support a series of spatially varying zero-energy deformations.

In general, the energy cost for a elastic deformation, i.e., the elastic energy, is a functional of the strain tensor. To the leading order, the elastic energy is

$$E = \int \mathbf{dr}\ c_{ijkl} \epsilon_{ij}(\mathbf{r}) \epsilon_{kl}(\mathbf{r}), \tag{4}$$

where c_{ijkl} are elastic constants. We have assumed that the elastic medium has no internal stress. This form for elastic energy is a standard description for an elastic medium. For an isotropic medium, these elastic constants reduces into two independent ones, bulk and shear moduli. Here, because we are considering a generic system, we will maintain this general form and allow the elastic constants to be independent. Same as above, here we adopt the Einstein summation convention, so all repeated indices are summed over. The higher order terms, which are not shown in Eq. (4), contain both higher order terms of the strain tensor as well as spatial derivatives on the strain tensor. Here, we will first ignore these higher order terms and their contributions will be examined in Sec. D.

If an elastic medium has (at least) one uniform deformation, which can be written as a position-independent strain tensor $\tilde{\boldsymbol{\epsilon}}$, that costs no elastic energy, we have

$$E = \int \mathbf{dr}\ c_{ijkl} \tilde{\epsilon}_{ij} \tilde{\epsilon}_{kl} = 0. \tag{5}$$

Because $\tilde{\boldsymbol{\epsilon}}$ is position independent, this indicates

$$c_{ijkl}\tilde{\epsilon}_{ij}\tilde{\epsilon}_{kl} = 0. \tag{6}$$

Next, we search for additional spatially varying zero-energy deformations in this system. It is easy to verify that a deformation described by the following strain tensor

$$\epsilon_{ij}(\mathbf{r}) = \tilde{\epsilon}_{ij}\phi(\mathbf{r}), \tag{7}$$

where $\phi(\mathbf{r})$ is an arbitrary scalar function, has zero elastic energy,

$$E = \int \mathbf{dr}\; c_{ijkl}\epsilon_{ij}(\mathbf{r})\epsilon_{kl}(\mathbf{r}) = c_{ijkl}\tilde{\epsilon}_{ij}\tilde{\epsilon}_{kl}\int \mathbf{dr}\; \phi(\mathbf{r})^2 = 0, \tag{8}$$

where we have used the fact that $c_{ijkl}\tilde{\epsilon}_{ij}\tilde{\epsilon}_{kl} = 0$ [Eq. (6)].

C. Constraints on the function $\phi(\mathbf{r})$ from curvature

It is important to point out that although the elastic energy [Eq. (8)] vanishes for any arbitrary function $\phi(\mathbf{r})$, not every function $\phi(\mathbf{r})$ corresponds to an elastic deformation. This is because the strain tensor is not an arbitrary rank-2 tensor. According to the definition of the strain tensor, in order to ensure that a strain tensor indeed describes a physical deformation, there has to exist a deformation $\mathbf{R}(\mathbf{r})$ such that

$$\epsilon_{ij}(\mathbf{r}) = (\partial_i R_k \partial_j R_k - \delta_{ij})/2, \tag{9}$$

is satisfied. This condition enforces strong strain constraints on the function $\phi(\mathbf{r})$ and in this section we will find the necessary and sufficient condition to guarantee a physical zero-energy deformation.

For this purpose, it is more convenient to use the metric tensor instead, which relates to the strain tensor through Eq. (3). The question now translates to finding the criterion, under which a metric tensor corresponds to a real physical deformation, i.e. to decided whether or not there exists a deformation $\mathbf{R}(\mathbf{r})$ exist such that

$$g_{ij}(\mathbf{r}) = \partial_i R_k \partial_j R_k \tag{10}$$

is satisfied. The answer to this question has been revealed in the study of differential geometry, where the same question is known as the problem of flat (local) coordinates. According to Riemann's Theorem, the necessary and sufficient condition for the existence of such an $\mathbf{R}(\mathbf{r})$ is that the metric tensor must have a zero curvature. The proof of this statement can be found in literature on Riemannian geometry or differential geometry. Here, instead of going through the full proof, we provide a physical picture to demonstrate the origin of this zero curvature condition. Because both our reference space and the target space (i.e. the material before and after the elastic deformation) are defined in a flat space, the mapping between these two spaces, $\mathbf{R}(\mathbf{r})$, must not have any nonzero curvature associated with it. Therefore, the metric tensor defined from this mapping must have zero curvature [1].

To determine the curvature for an arbitrary metric tensor $g_{ij}(\mathbf{r})$, we first define the Levi-Civita connection, i.e. the Christoffel symbols, using the derivative of g_{ij},

$$\Gamma_{kij} = \frac{1}{2}(\partial_j g_{ki} + \partial_i g_{kj} - \partial_k g_{ij}). \tag{11}$$

Then, by taking another derivative to the Levi-Civita connection, the Riemann curvature tensor is obtained,

$$R_{ijkl} = \partial_k \Gamma_{ilj} - \partial_l \Gamma_{ikj} + g^{mn}\Gamma_{ikm}\Gamma_{nlj} - g^{mn}\Gamma_{ilm}\Gamma_{nkj}, \tag{12}$$

where g^{mn} is the matrix inverse of the metric tensor g_{ij}.

For a physical deformation in a flat space, the Ricci curvature tensor must vanish, $R_{ijkl} = 0$. For the zero energy deformations shown in Eq. (7), the corresponding metric tenor is

$$g_{ij}(\mathbf{r}) = \tilde{\epsilon}_{ij}\phi(\mathbf{r}) + \delta_{ij}. \tag{13}$$

In 2D, generically, the function $\phi(\mathbf{r})$ depends on both coordinates x and y. However, the zero curvature condition enforces a constraint on $\phi(\mathbf{r})$. Using Eq. (12) it is straightforward to verify that the curvature vanishes, if and only if $\phi(\mathbf{r})$ takes one of the following two forms

$$\phi(\mathbf{r}) = f_+(x + \lambda_+ y) \tag{14}$$

or

$$\phi(\mathbf{r}) = f_-(x + \lambda_- y) \tag{15}$$

Here, $f_+(s)$ and $f_-(s)$ are arbitrary functions of s. and λ_+ and λ_- are two constants that are determined by the strain tensor of the uniform zero-energy deformation

$$\lambda_+ = (\tilde{\epsilon}_{xy} + \sqrt{-\det\tilde{\boldsymbol{\epsilon}}})/\tilde{\epsilon}_{xx}, \tag{16}$$

$$\lambda_- = (\tilde{\epsilon}_{xy} - \sqrt{-\det\tilde{\boldsymbol{\epsilon}}})/\tilde{\epsilon}_{xx}, \tag{17}$$

where $\det\tilde{\boldsymbol{\epsilon}}$ is the determinant of $\tilde{\boldsymbol{\epsilon}}$. It is worth pointing out that this result is independent of the choice of the coordinate. If the directions of x, y are chosen differently, $\lambda_\pm$ will change accordingly, but the two directions given by $x + \lambda_\pm y$ are invariant.

We have shown in Eq. (8) that these deformations cost no elastic energy. Because the zero curvature condition is the necessary and sufficient condition which guarantees that the strain tensor defined in Eq. (7) corresponds to a physical deformation, we conclude that the following spatially varying deformations are all zero energy modes of the system

$$\begin{aligned}\epsilon_{ij}(\mathbf{r}) &= \tilde{\epsilon}_{ij} f_+(x + \lambda_+ y),\\ \epsilon_{ij}(\mathbf{r}) &= \tilde{\epsilon}_{ij} f_-(x + \lambda_- y).\end{aligned} \tag{18}$$

Because we can choose arbitrary f_+ and f_-, the number of these zero energy deformations is infinite in the continuous theory. In a real system, with lattice structure and with finite size, the number of zero modes scales with the linear size of the system $\sim L/a$, where L is the size of the system and a is the lattice constant. Thus the number of these zero modes is sub-extensive.

In summary, we prove here that for a 2D elastic system, as long as there exists one uniform zero-energy mode, which is described by a spatially independent strain tensor $\tilde{\boldsymbol{\epsilon}}$, there must exist two families of spatially varying zero-energy modes, as shown in Eq. (18).

D. Higher order terms in the elastic energy

In our analysis above, we ignored higher order terms in the elastic energy. These higher order terms involve both higher powers in $\boldsymbol{\epsilon}$ and higher order derivatives, such as $\partial\boldsymbol{\epsilon}$.

In the previous section we solved for modes that have zero elastic energy in the leading order theory. Restoring contributions from higher order terms, the elastic energy of these modes is

$$E = 0 + O(\boldsymbol{\epsilon}^3) + O(\partial\boldsymbol{\epsilon}\partial\boldsymbol{\epsilon}), \tag{19}$$

which is small when the strain is small and slowly varying in space. Thus, strictly speaking, these zero modes should be called floppy modes because they are not necessarily exactly zero energy.

In addition, in the Article, we consider frequencies of plane waves (in the bulk or on the surface) that belong to these two families of floppy modes with wave number k. Our theory then predicts that the frequency of these waves are

$$\omega = O(k^2). \tag{20}$$

Ordinary plane waves in stable elastic medium have $\omega = c\,k$, where c is the speed of sound. In contrast, these floppy modes correspond to plane waves with zero speed of sound.

This zero sound velocity is a key signature of the systems with floppy uniform deformations that we study here. Regardless of the details of the system, these conclusions hold universally.

In special families of structures with uniform floppy twisting (e.g., Maxwell lattices), these floppy modes may have exactly zero elastic energy, even if higher order terms are taken into account. This phenomenon is discussed in our Article, where we show that the exact zero elastic energy is protected by Maxwell's counting rule. Nevertheless, it is worthwhile to emphasize that although in the general case (where there is no protection from the counting rule) the elastic energy receives higher order corrections, the acoustic sound velocity for these modes will always be zero.

SUPPLEMENTARY NOTE 5: DOMAIN SOFT TWISTINGS

As we have discussed, periodic Maxwell lattices can undergo smooth, zero-energy transformations under generalized twisting coordinate θ, which transforms their primitive vectors. The magnitudes of the primitive vectors vary smoothly and are bounded, and hence have local maxima and minima. Only at such local maxima and minima does the Guest mode (uniform soft twisting) not couple to uniaxial strain along the lattice direction, and hence there are self stresses present along these lines. These are precisely the configurations discussed in the main text in which lines of linear zero modes enter the bulk. However, these may also be extended to nonlinear zero modes. Previously, we considered a uniform lattice in which every unit cell underwent the same deformation parametrized by θ. Now let us instead consider a lattice in which configurations may differ between individual crystal cells but which nevertheless repeat periodically:

$$\theta_{i,j} = \theta_{i,j+L} = \theta_{i+L,j}, \tag{21}$$

where i, j is the cell index, L is the number of cells along one side of the system, and $\theta_{i,j}$ is the cell's configuration coordinate. Consider, in particular, a configuration in which the coordinate is uniform for all j along a row but differs for rows i and $i+1$, as depicted in figures in the main text. A necessary and, for Maxwell lattices, sufficient condition to ensure that these two rows may meet without stretching any bonds is simply that the primitive vector along the interface be equal in length (differences in orientation may be met by rotating one of the rows relative to the other):

$$|\mathbf{a_1}(\theta_{i,j})| = |\mathbf{a_1}(\theta_{i+1,j})|. \tag{22}$$

As shown in Fig. S1, this condition may be satisfied not only by the uniform solution but one in which two subsequent domains have distinct configurations (diagrams of such lattices are shown in the main text). At the critical points a domain structure may be induced in a previously uniform material. At such a point, the system is necessarily homogenous, as there is a unique maximum (or minimum, which generally involves self-intersecting configurations). However, each row separately may twist in a positive or negative direction. To linear order, these are simply the L bulk zero modes of the lattice with N_y rows, but this formulation shows that they may be extended to the nonlinear regime. Hence, there are 2^L different uniform soft twistings accessible from a critical point. The point is "critical" not just in the sense of altering the topological polarization, but in determining which of the many nonlinear elastic instabilities will be induced. However, once the domain structure is induced and the system is twisted away from the critical point there is once again only a single global mode that preserves the periodic boundary conditions, the Domain Soft Twisting.

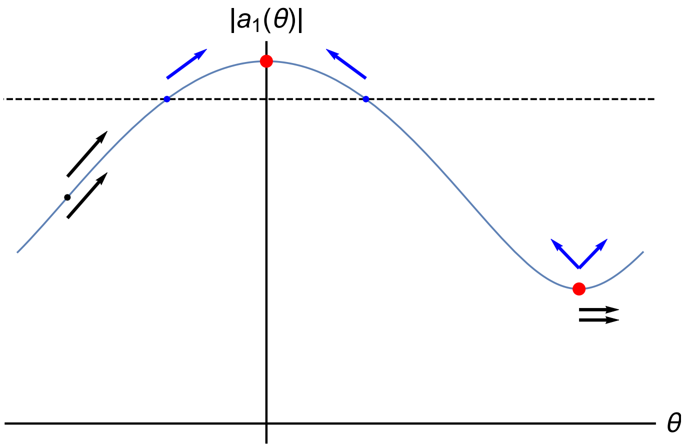

Supplementary Figure 2. **Change in the magnitude of the first primitive vector under soft twisting.** Points and arrows denote the allowed configurations and transformations of adjacent domains. The leftmost (black) point represents two identical regions which twist in the same manner. The blue points connected via the dashed line represent adjacent domains which require equal magnitudes of their primitive vectors, hence the soft twisting carries them in opposite directions. Only at critical points (red) where the magnitude has local extrema can domain structure be induced or removed—here there is uniform soft twisting (black arrows below point) and domain soft twisting (blue arrows above point).

For the deformed kagome lattice, the soft twistings in the two domains have a particularly simple configuration. The primitive vector is simply the sum of two vectors $\mathbf{r}_1, \mathbf{r}_2$ along sides of the triangular tiles. The twisting coordinates θ_t, θ_b in the two domains each rotate the two vectors relative to one another. Hence, the condition of preserving the length of the primitive vector is, in terms of a rotation matrix $\mathbf{R}(\theta_{t(b)} - \theta_{\mathbf{a_1}})$ simply

$$|\mathbf{r}_1 + \mathbf{R}(\theta_t - \theta_{\mathbf{a_1}})\mathbf{r}_2| = |\mathbf{r}_1 + \mathbf{R}(\theta_b - \theta_{\mathbf{a_1}})\mathbf{r}_2| \\ \Rightarrow \cos(\theta_t - \theta_{\mathbf{a_1}}) = \cos(\theta_b - \theta_{\mathbf{a_1}}), \tag{23}$$

where we have used the fact that the two edges are collinear at the critical configuration. Hence, for the deformed kagome the only two possible types of domains in the $\mathbf{a_1}$ direction satisfy

$$\theta_t - \theta_{\mathbf{a_1}} = -(\theta_b - \theta_{\mathbf{a_1}}), \tag{24}$$

as shown in Fig. 6 in the main text.

More generally, there is no reason a Maxwell lattice cannot have more complicated configurations in which there are several compatible twisting angles and more varied domain structures are possible. However, it is not generally possible to have domain structure along two lattice conditions simultaneously, since Supplementary Equation (22) fixes the relation between two subsequent twisting coordinates, with no additional degrees of freedom available to satisfy the analogous condition in an additional lattice direction.

[2] J. C. Maxwell, Philos. Mag. **27**, 294 (1864).
[3] C. Calladine, Int. J. Solids Struct. **14**, 161 (1978).
[4] M. Thorpe, J. Non-Cryst. Solids **57**, 355 (1983).
[5] A. J. Liu, S. R. Nagel, W. van Saarloos, and M. Wyart, in *Dynamical heterogeneities in glasses, colloids, and granular media*, edited by L. Berthier, G. Biroli, J.-P. Bouchaud, L. Cipeletti, and W. van Saarloos (Oxford University Press, 2010) Chap. 9, pp. 298–340.
[6] X. Mao, N. Xu, and T. C. Lubensky, Phys. Rev. Lett. **104**, 085504(1 (2010).
[7] T. C. Lubensky, C. Kane, X. Mao, A. Souslov, and K. Sun, Reports on Progress in Physics **78**, 073901(1 (2015).
[8] S. D. Guest and J. W. Hutchinson, J. Mech. Phys. Solids **51**, 383 (2003).
[9] K. D. Hammonds, M. T. Dove, A. P. Giddy, V. Heine, and B. Winkler, American Mineralogist **81**, 1057 (1996).
[10] J. S. Evans, Journal of the Chemical Society, Dalton Transactions , 3317 (1999).
[11] G. N. Greaves, A. L. Greer, R. S. Lakes, and T. Rouxel, Nat. Mater. **10**, 823 (2011).
[12] R. Gatt, L. Mizzi, J. I. Azzopardi, K. M. Azzopardi, D. Attard, A. Casha, J. Briffa, and J. N. Grima, Scientific reports **5** (2015).
[1] T. Frankel, *The geometry of physics: an introduction* (Cambridge University Press, 2011).
